# Essential yet Ill-defined: leadership roles to support fourth-year medical students in pediatrics

**DOI:** 10.1080/10872981.2021.1950108

**Published:** 2021-07-07

**Authors:** Molly Rideout, Marie Dawlett, Jennifer Plant, Maribeth Chitkara, Jennifer L. Trainor

**Affiliations:** a Department of Pediatrics, Larner College of Medicine at the University of Vermont, Burlington, VT, USA; b Department of Pediatrics, University of Texas Medical Branch School of Medicine, Galveston, TX, USA; c Department of Pediatrics, University of California Davis School of Medicine, Sacramento, CA, USA; d Department of Pediatrics, Renaissance School of Medicine at Stony Brook University, NY, USA; e Department of Pediatrics, Northwestern University, Feinberg School of Medicine, Chicago, IL, USA

**Keywords:** Fourth year pediatrics director, final year medical school, medical student education leadership, transition to residency pediatrics, fourth year medical school

## Abstract

Background: Few studies have been published about specialty-specific fourth-year medical student leadership in any discipline. This paper provides insight from pediatric educators about the current status and recommendations for pediatric-specific fourth-year leaders. Objective: To identify the prevalence of pediatric fourth-year medical student directors across the US and Canada and to compare current and ideal responsibilities for this role. Methods: Five multi-part questions were written and submitted for the 2019 Council on Medical Student Education in Pediatrics (COMSEP) Annual Survey and subsequently disseminated to all COMSEP member physicians. Anonymous responses were collected and results analyzed. The study was IRB exempt. Results: The program-level survey response rate was 79%. Of 115 respondent medical schools, 37% reported having a pediatric fourth-year director separate from the clerkship director, with an average of 9.8% full-time equivalent (FTE) protected time for the role. In contrast, individuals indicated 20% FTE would be ideal for fourth-year director responsibilities. The most common role identified for pediatric fourth-year directors was directing sub-internships. Respondents indicated it would be ideal for pediatric fourth-year directors to have an increased level of involvement in all areas queried in the survey, especially directing a pediatric residency preparatory course/boot camp, faculty development for educators of fourth-year students, and remediating fourth-year students. Conclusions: As specialty-specific experiences have grown in the fourth year of medical school, there is an increasing demand for faculty leadership separate from direction of the pediatric clerkship. In this national survey, pediatric educators expressed a need for additional protected time to lead fourth-year specific activities. Similar findings in other disciplines would support advocating for more protected time and expanded roles for specialty-specific fourth-year directors nationally.

## Introduction

Although formal recommendations have been made about fourth-year curricular content [1], no published guidelines exist to inform the approach to fourth-year curricular leadership, and responsibilities and protected time vary greatly across institutions. In contrast, standardized roles and responsibilities for clerkship directors have been developed [2,3], including a recommendation for faculty protected time [4]. Responsibilities for directing pediatric fourth-year activities are substantial, including significant advising requirements, coaching during sub-internships, and direction of residency preparatory courses [5–14]. This study is an attempt to clarify the ***current*** responsibilities and protected time for pediatric fourth-year directors across the US and Canada as well as elicit the opinions of faculty regarding ***ideal*** roles and protected time for this position. For the purposes of this study, the term ‘fourth year’ refers to the post-clerkship phase of medical school, and ‘specialty-specific fourth-year director’ refers to a leader of the fourth-year medical student program within a specialty (in this case, pediatrics).

## Methods

Survey questions regarding the leadership of the pediatric fourth year were included in the 2019 Council on Medical Student Education in Pediatrics (COMSEP) Annual survey, a survey addressing specific areas of interest to the pediatric education community. We drafted five survey questions based on input from a multi-institutional group of pediatric fourth-year directors on the Curriculum Task Force of COMSEP and then revised them iteratively based on expert review and pilot testing at the authors’ institutions. The COMSEP Annual Survey Committee selected the submitted questions as part of a competitive process, provided blinded peer feedback, and performed additional pilot testing. The study was deemed exempt by the IRB at the University of Vermont.

Survey questions (shown in Appendix A) included whether a program had a pediatric fourth-year director separate from the clerkship director, and if so, the **current**amount of protected time available and **current** roles of the position (using a 4-point Likert scale [not at all/somewhat/mostly/completely responsible] to indicate actual level of responsibility). Additional questions asked about the **ideal** amount of protected time for directing the pediatric fourthyear and the **ideal** roles for the position (using a 4-point Likert scale [not at all/somewhat/mostly/completely responsible] to indicate ideal level of responsibility).

Demographic data was also collected and linked to the survey data by unique identifiers. COMSEP disseminated the survey by email to all COMSEP member physicians in March 2019 via a personalized link.

**Figure 1.**
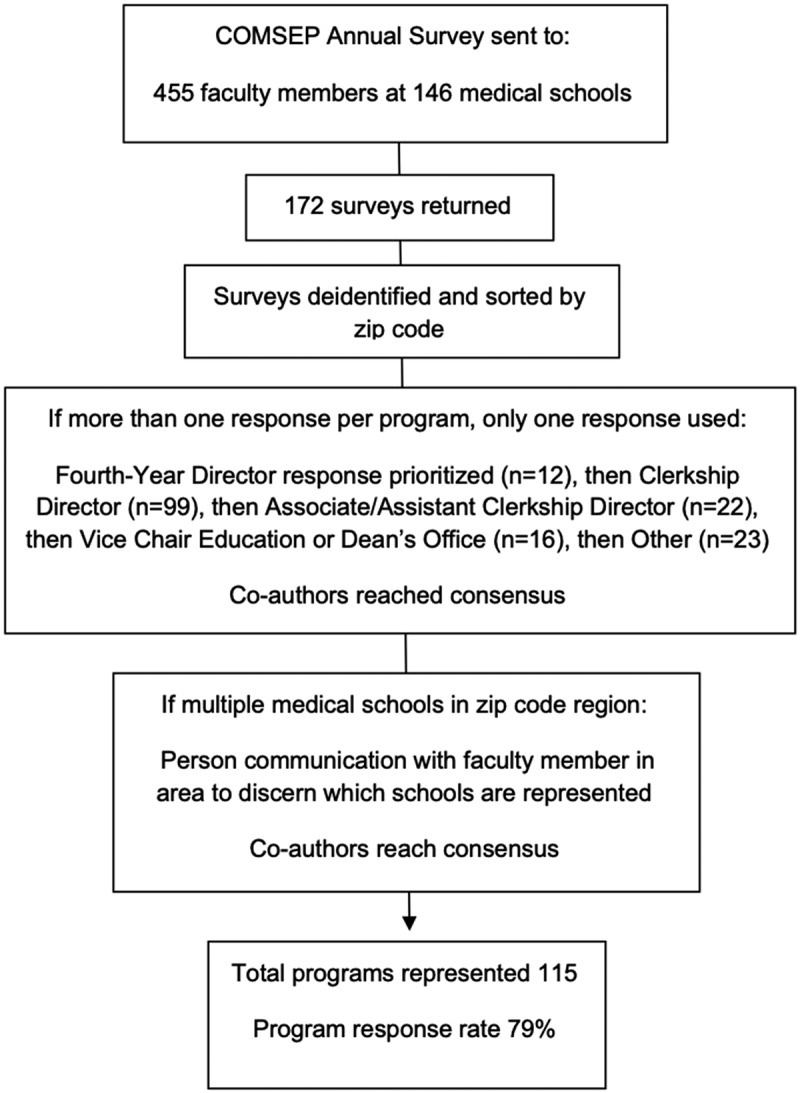
Caption: Process for obtaining program-level data

We used descriptive statistics to analyze the data, including the mean protected time for the position and the frequency of different roles for the position, both current and ideal. To determine the degree of involvement of different roles, we combined ‘mostly’ and ‘completely’ responsible answers for each role and categorized this as ‘High Level of Responsibility’ (see Table 1). We determined program-level responses by sorting deidentified surveys by zip code. For programs with multiple survey respondents, we selected one response per program using the following priority order (based on presumed familiarity with fourth-year leadership, by author consensus): fourth-year director, clerkship director, associate clerkship director, Dean’s office faculty, other faculty. (Figure 1) If respondents had identical roles, we prioritized respondents with more years of experience. We also analyzed responses within programs for level of discordance.

**Table 1.** Percent of programs indicating that the fourth-year director at their institution has most or complete responsibility for various roles, based on a 4-point Likert scale

	The *current* fourth-year director is mostly/completely responsible^a^ for the following roles:	A fourth-year director should *ideally* be mostly/completely responsible^a^ for the following roles:
Directing Pediatric Sub-I’s	73	85
Directing Pediatric Electives	39	61
Advising for residency interviews/applications	31	59
Advising for careers in pediatrics	31	55
Providing faculty development to support fourth-year education	29	69
Identifying fourth-year students not meeting standards for performance	41	68
Remediating fourth-year students not meeting standards for performance	25	69
Directing a residency prep course (boot camp)	24	66

*^a^4-point Likert scale not at all/somewhat/mostly/completely responsible*.

## Results

The 2019 COMSEP Annual Survey was sent to 455 COMSEP member physicians from 146 medical schools. One hundred seventy-two respondents representing 115 unique medical schools (109 from United States/6 from Canada) completed the survey, for a program-level response rate of 79%. Of programs responding, 37% reported having a dedicated pediatric fourth-year director separate from the clerkship director and 63% did not. The mean protected FTE for this role at programs with a dedicated director was 9.8% (mean/mode 10%/range 0–30). In contrast, respondents reported the ***ideal*** mean protected FTE for the role was 20% (median/mode 20%/range 0–74).

Roles for fourth-year directors varied greatly (Table 1). For programs with directors, the most common role which included a high level of responsibility was directing pediatric sub-internships. Respondents chose an ideal level of responsibility that was higher than the current level for all roles listed, with the largest gap for faculty development, remediating students, and directing a residency preparatory course.

There were 35/115 programs with multiple respondents; the responses were prioritized and one response per program used for data analysis. The remaining repsonses for each program were analyzed for discordance; 12 of these programs had discordant responses regarding the presence of a fourth-year director and 7 regarding current roles, while 11 programs had responses about current FTE that differed by more than 5%.

## Discussion

In our study, pediatric educators noted that ideally there would be increased protected time for fourth-year director duties and increased involvement for this role in all areas, particularly in directing a residency preparatory course for graduating students, remediating struggling students, and providing faculty development for others involved in post-clerkship medical student education. These findings are not surprising given the many student needs and programmatic challenges that have been described in the literature about this final phase of medical school training.

The fourth year of medical school remains a controversial curricular challenge for medical educators [15] whose chief purpose is to ensure that students successfully complete their undergraduate medical education whilst confidently transitioning to residency [11]. Concerns outlined in the literature about the fourth year include a lack of clarity about the overall purpose, problems regarding curricular organization, and issues with the educational quality of offered courses [10–13, 15,16]. While often criticized by students for a lack of cohesion, curricular integration and adequate residency preparation, the fourth year is likewise embraced by them for its flexibility and individualization of course selection, opportunity for career exploration and participation in scholarly activities to enhance residency applications [12]. A majority of students agree that the fourth year is a time for residency selection and preparation, and place a more significant weight on extrinsic goals such as residency selection and preparation than intrinsic ones such as personal growth, reflection and development [13]. Fourth-year directors, with their potential involvement in course development and administration as well as student mentoring and coaching, have the potential to address some of these issues and help guide students through this very important and individuzalized experience [16].

The literature also supports the idea that fourth-year directors with specialty-specific expertise are essential in advising students. In a training environment that is becoming increasingly competitive and unpredictable in the wake of the COVID-19 pandemic, it is imperative that institutions identify specialty-specific fourth-year directors who are able to provide a holistic approach to navigating the residency application process [14]. Advising students as they map out their path to a career in pediatrics requires an approach that integrates efforts of student affairs staff, clerkship directors, career advisors and teaching faculty [17]. General advisors within institutions may have insight into application process and AAMC guidelines, but not necessarily in-depth specialty-specific expertise.

The provision of specialty-specific residency preparedness training for graduating students is another essential role for fourth-year directors that has been recognized in the literature. The AAMC core entrustable professional activies (EPAs) provide a scaffolding of achievable skills for trainees and program directors to reference when determining overall preparedness for residency [18], and studies have demonstrated large discrepancies in graduating medical students’ confidence regarding specific EPAs and the perception of program directors [19] as well as gaps in expected and observed performance regarding EPAs [20] . When fourth-year students are surveyed about preparedness for residency, however, they most frequently identify the competency domain of medical knowledge as needing reinforcement over others such as professionalism and interpersonal communication [21]. Proponents of longitudinal course structures in the fourth year of medical school cite the benefit of more valuable and accurate assessments of learners using this approach [10]. A specialty-specific fourth-year director could narrow the gap between perceived and observed performance by using EPA- and core competency-based frameworks with senior trainees longitudinally, both in clinical coursework and scholarship, during this transitional period. Although pediatric educators expressed that ideally there would be an increase in protected time for this role in this survey-based study, the financial implications of the role of specialty-specific fourth-year directorship is important to consider. Whether the financial support for the position would originate at a departmental or medical school level is not clear, but the authors of this study believe it is an essential role worthy of institutional support. Clarifying the structure and responsibilities of the specialty-specific fourth-year director role and ensuring faculty have the time to perform this role is a first step in establishing standard recommendations.

This study has limitations typical of a survey-based design, including sampling bias. Also, in order to establish program-level data, responses were prioritized through a consensus-driven systematic method, but there were discordant responses within some programs, likely related to variability of awareness of the current status of fourth-year leadership. Similar studies in other disciplines would support advocating for protected time for specialty-specific fourth-year leadership, and this oversight may prove beneficial for a successful UME to GME transition.

## Appendix A

**Survey Questions**:

Q1 Is there a dedicated pediatric fourth-year director at your institution, separate from the clerkship director? (yes/no)

Q2 To what extent is the fourth-year director responsible for the following duties^1^ at your institution? (not at all, somewhat, mostly, completely, n/a)

Q3 Ideally, to what extent should a pediatric fourth-year director be responsible for the following duties^1^? (not at all, somewhat, mostly, completely, n/a)

Q4 With regard to protected time for directing the pediatric fourth year at your institution (separate from clerkship director duties), what percent FTE is provided?

Q5 With regard to protected time for directing the pediatric fourth year (separate from clerkship director duties), what percent FTE would be ideal?

**^1.^*Potential duties for fourth-year directors in pediatrics***:

*Directing pediatric sub-internships*

*Directing pediatric electives*

*Advising for residency interviews and applications*

*Advising for careers in pediatrics*

*Providing faculty development to support fourth year education*

*Identifying fourth year students not meeting standards for performance*

*Remediating fourth year students not meeting standards for performance*

*Directing a residency preparatory course (boot camp)*
